# On Certain Subclass of Meromorphic Spirallike Functions Involving the Hypergeometric Function

**DOI:** 10.1155/2014/541371

**Published:** 2014-05-25

**Authors:** Lei Shi, Zhi-Gang Wang

**Affiliations:** School of Mathematics and Statistics, Anyang Normal University, Anyang, Henan 455000, China

## Abstract

We introduce and investigate a new subclass *M*_*m*_^1^(*θ*, *λ*, *η*) of meromorphic spirallike functions. Such results as integral representations, convolution properties, and coefficient estimates are proved. The results presented here would provide extensions of those given in earlier works. Several other results are also obtained.

## 1. Introduction

Let *M* denote the class of functions *f* of the form

(1) $$\begin{array}{c} f\left(z\right)=\frac{1}{z}+\overset{\infty }{\underset{n=1}{\sum }}a_{n}z^{n}, \end{array}$$

which are analytic in the punctured open unit disk:

(2) $$\begin{array}{c} U^{*}:=\left\{z:z\in C,\;\;0<\left|z\right|<1\right\}=:U\setminus \left\{0\right\}. \end{array}$$

Let *f*, *g* ∈ *M*, where *f* is given by (1) and *g* is defined by

(3) $$\begin{array}{c} g\left(z\right)=\frac{1}{z}+\overset{\infty }{\underset{n=1}{\sum }}b_{n}z^{n}. \end{array}$$

Then the Hadamard product (or convolution) *f*∗*g* of *f* and *g* is defined by

(4) $$\begin{array}{c} \left(f*g\right)\left(z\right)=\frac{1}{z}+\overset{\infty }{\underset{n=1}{\sum }}a_{n}b_{n}z^{n}:=\left(g*f\right)\left(z\right). \end{array}$$

Let *P* denote the class of functions *p* given by

(5) $$\begin{array}{c} p\left(z\right)=1+\overset{\infty }{\underset{n=1}{\sum }}p_{n}z^{n}\;\left(z\in U\right), \end{array}$$

which are analytic in *U* and satisfy the condition

(6) $$\begin{array}{c} R\left(p\left(z\right)\right)>0\;\left(z\in U\right). \end{array}$$

For *θ* which is real with |*θ*| < *π*/2, 0 ≤ *γ* < 1, we denote by MS*(*θ*, *γ*) and MK*(*θ*, *γ*) the subclasses of *f* ∈ *M* which are defined, respectively, by

(7) $$\begin{array}{c} R\left(e^{i\theta }\frac{zf^{\prime }\left(z\right)}{f\left(z\right)}\right)<-\gamma \cos\theta \;\left(z\in U^{*}\right),\\ R\left(e^{i\theta }\frac{{\left(zf^{\prime }\left(z\right)\right)}^{\prime }}{f^{\prime }\left(z\right)}\right)<-\gamma \cos\theta \;\left(z\in U^{*}\right). \end{array}$$

By setting *θ* = 0 in (7), we get the well-known subclasses of *f* ∈ *M* consisting of meromorphic functions which are starlike and convex of order *γ*  (0 ≤ *γ* < 1), respectively. For some recent investigations on meromorphic spirallike functions and related topics, see, for example, the earlier works [1–4] and the references cited therein.

For *η* > 1, Wang et al. [5] and Nehari and Netanyahu [6] introduced and studied the subclass *M*(*η*) of *M* consisting of functions *f* satisfying

(8) $$\begin{array}{c} R\left(\frac{zf^{\prime }\left(z\right)}{f\left(z\right)}\right)>-\eta \;\left(z\in U^{*}\right). \end{array}$$

Let *A* be the class of functions of the form

(9) $$\begin{array}{c} f\left(z\right)=z+\overset{\infty }{\underset{n=1}{\sum }}a_{n}z^{n}, \end{array}$$

which are analytic in *U*. A function *f* ∈ *A* is said to be in the class S*(*θ*, *δ*, *γ*) if it satisfies the condition

(10) R(eiθzf′(z)(1−δ)f(z)+δzf′(z))>γcos⁡⁡θ(z∈U;  |θ|<π2;  0≤δ<1;  0≤γ<1).

The function class S*(*θ*, *δ*, *γ*) is introduced and studied recently by Orhan et al. [7]. An analogous of the class S*(*θ*, *δ*, *γ*) has been studied by Murugusundaramoorthy [8].

For complex parameters *α*_1_,…, *α*_*l*_ and *β*_1_,…, *β*_*m*_  (*β*_*j*_ ≠ 0, −1, −2,…; *j* = 1,2,…, *m*) the generalized hypergeometric function _*l*_ *F*_*m*_(*z*) is defined by

(11) Flm(z)≡Flm(α1,…,αl;β1,…,βm;z):=∑n=0∞(α1)n⋯(αl)n(β1)n⋯(βm)nznn!(l≤m+1;  l,m∈N0:=N∪{0};  z∈U),

where *N* denotes the set of all positive integers and (*a*)_*k*_ is the Pochhammer symbol defined by

(12) $$\begin{array}{c} {\left(a\right)}_{n}=\{\begin{array}{cc} 1, & n=0,\\ a\left(a+1\right)\left(a+2\right)\cdots \left(a+n-1\right), & n\in N;\;\;a\in C. \end{array} \end{array}$$

For a function *f* ∈ *M*, we consider a linear operator (which is a meromorphically modified version of the familiar Dziok-Srivastava linear operator [9, 10]:

(13) Hml[α,β]f(z)=H(α1,…,αl;β1,…,βm)f(z)=[z−1lFm(α1,…,αl;β1,…,βm;z)] ∗f(z),

where *α*_*s*_ > 0, *β*_*t*_ > 0  (*s* = 1,…, *l*; *t* = 1,…, *m*; *l* ≤ *m* + 1; *l*, *m* ∈ *N*_0_).

From the definition of the operator *H*_*m*_^*l*^[*α*, *β*]*f*, it is easy to observe that

(14) $$\begin{array}{c} H_{m}^{l}\left[\alpha ,\beta \right]f\left(z\right)=\frac{1}{z}+\overset{\infty }{\underset{n=1}{\sum }}\phi_{n}\left(\alpha ,\beta ;l;m\right)a_{n}z^{n}, \end{array}$$

where

(15) $$\begin{array}{c} \phi_{n}\left(\alpha ,\beta ;l;m\right)=\frac{{\left(\alpha_{1}\right)}_{n+1}\cdots {\left(\alpha_{l}\right)}_{n+1}}{{\left(\beta_{1}\right)}_{n+1}\cdots {\left(\beta_{m}\right)}_{n+1}}\frac{1}{\left(n+1\right)!} \end{array}$$

is a positive number for all *n* ∈ *N*.

Recently, Aouf [11], Liu and Srivastava [12], and Raina and Srivastava [13] obtained many interesting results involving the linear operator *H*_*m*_^*l*^[*α*, *β*]. In particular, for

(16) $$\begin{array}{c} l=2,\;\;m=1,\;\;\alpha_{1}=a,\;\;\beta_{1}=c,\;\;\alpha_{2}=1, \end{array}$$

we obtain the following linear operator:

(17) $$\begin{array}{c} L\left(a,c\right)f\left(z\right):=H\left[\alpha_{1},1;\beta_{1}\right]f\left(z\right)\;\left(z\in U^{*}\right), \end{array}$$

which was introduced and investigated earlier by Liu and Srivastava [14] and was further studied in a subsequent investigation by Srivastava et al. [15]. It should also be remarked that the linear operator *H*_*m*_^*l*^[*α*, *β*] is a generalization of other linear operators considered in many earlier investigations (see, e.g., [16–18]).

Using the operator *H*_*m*_^*l*^[*α*, *β*]*f*, we introduce the following class of meromorphic functions.

**Definition 1**

For |*θ*| < *π*/2, 0 ≤ *λ* < 1/2, and *η* > 1, let *M*_*m*_^*l*^(*θ*, *λ*, *η*) denote a subclass of *M* consisting of functions satisfying the condition that

(18) R(eiθ(1−2λ)z(Hml[α,β]f(z))′(1−λ)Hml[α,β]f(z)+λz(Hml[α,β]f(z))′)  >−ηcos⁡⁡θ, (z∈U∗),

where *H*_*m*_^*l*^[*α*, *β*]*f* is given by (13).

We note that, for *l* = 2, *m* = 1, *α*_1_ = *α*_2_ = 1, *β*_1_ = 1, and *θ* = *λ* = 0, the class *M*_1_^2^(0,0, *η*) becomes the class *M*(*η*).

In the present paper, we aim at proving some interesting properties such as integral representations, convolution properties, and coefficient estimates for the class *M*_*m*_^*l*^(*θ*, *λ*, *η*).

The following lemma will be required in our investigation.

**Lemma 2**

Suppose that the sequence {*A*_*n*_}_*n*=1_^*∞*^ is defined by

(19) A1=(1−2λ)(η−1)cos⁡θ(1−λ)ϕ1(α,β;l;m),An+1=2(η−1)cos⁡θ(n+2)(1−λ)ϕn+1(α,β;l;m)×[1−2λ+∑k=1nϕk(α,β;l;m)(1−λ+kλ)Ak].

Then

(20) An=(1−2λ)(η−1)cos⁡θ(1−λ)nϕn(α,β;l;m) ×∏k=1n−1(k+1)(1−λ)+2(1−λ+kλ)(η−1)cos⁡θk+2,                       (n≥2).

**Proof**

From (19), we have

(21) (n+2)(1−λ)ϕn+1(α,β;l;m)An+1 =2(η−1)cos⁡θ[1−2λ          +∑k=1nϕk(α,β;l;m)(1−λ+kλ)Ak],(n+1)(1−λ)ϕn(α,β;l;m)An =2(η−1)cos⁡θ[1−2λ          +∑k=1n−1ϕk(α,β;l;m)(1−λ+kλ)Ak].

Combining (21), we find that

(22) An+1An=(n+1)(1−λ)+2(1−λ+nλ)(η−1)cos⁡θ(n+2)(1−λ) ·ϕn(α,β;l;m)ϕn+1(α,β;l;m).

Thus, for *n* ≥ 2, we deduce from (22) that

(23) An=AnAn−1⋯A3A2·A2A1·A1=n(1−λ)+2(1−nλ)(η−1)cos⁡θ(n+1)(1−λ)⋯ ×3(1−λ)+2(1+λ)(η−1)cos⁡θ4(1−λ) ·2(1−λ)+2(η−1)cos⁡θ3(1−λ) ·ϕn−1(α,β;l;m)ϕn(α,β;l;m)⋯ϕ2(α,β;l;m)ϕ3(α,β;l;m) ·ϕ1(α,β;l;m)ϕ2(α,β;l;m)·(1−2λ)(η−1)cos⁡θ(1−λ)ϕ1(α,β;l;m)=(1−2λ)(η−1)cos⁡θ(1−λ)nϕn(α,β;l;m) ×∏k=1n−1(k+1)(1−λ)+2(1−λ+kλ)(η−1)cos⁡θk+2.

This completes the proof of Lemma 2.

## 2. Main Results

We begin by proving the following integral representation for the class *M*_*m*_^*l*^(*θ*, *λ*, *η*).

**Theorem 3**

Let *f* ∈ *M*_*m*_^*l*^(*θ*, *λ*, *η*). Then

(24) f(z)=(z−1+∑n=1∞ϕn−1(α,β;l,m)zn) ∗[z−1·exp⁡(∫0z(((1−2λ)(η−1)            ×(1+e−2iθ)ω(t))           ×([(1−λ)(1−ω(t))             −λ(η−1)(1+e−2iθ)             ×ω(t)]t)−1)dt)],                   (z∈U∗),

where *ω* is analytic in *U* with *ω*(0) = 0 and |*ω*(*z*)| < 1.

**Proof**

Suppose that *f* ∈ *M*_*m*_^*l*^(*θ*, *λ*, *η*) and

(25) τ(z):=(((eiθ(1−2λ)z(Hml[α,β]f(z))′)   ×((1−λ)Hml[α,β]f(z)     +λz(Hml[α,β]f(z))′)−1)   +ηcos⁡θ+isinθ)×((η−1)cos⁡θ)−1,                   (z∈U).

We know that *τ* ∈ *P*, which implies

(26) (((eiθ(1−2λ)z(Hml[α,β]f(z))′)  ×((1−λ)Hml[α,β]f(z)+λz(Hml[α,β]f(z))′)−1)  +ηcos⁡θ+isinθ)×((η−1)cos⁡θ)−1  =1+ω(z)1−ω(z),

where *ω* is analytic in *U* with *ω*(0) = 0 and |*ω*(*z*)| < 1. We find from (26) that

(27) (1−2λ)z(Hml[α,β]f(z))′(1−λ)Hml[α,β]f(z)+λz(Hml[α,β]f(z))′  =−1+[1+(η−1)(1+e−2iθ)]ω(z)1−ω(z),

which follows

(28) (Hml[α,β]f(z))′Hml[α,β]f(z)+1z =(1−2λ)(η−1)(1+e−2iθ)ω(z)z[(1−λ)(1−ω(z))−λ(η−1)(1+e−2iθ)ω(z)].

Integrating both sides of (28) yields

(29) log⁡(zHml[α,β]f(z)) =∫0z(1−2λ)(η−1)(1+e−2iθ)ω(t)[(1−λ)(1−ω(t))−λ(η−1)(1+e−2iθ)ω(t)]tdt.

From (29), we obtain

(30) Hml[α,β]f(z) =z−1·exp⁡(∫0z(((1−2λ)(1+e−2iθ)(η−1)ω(t))           ×([(1−λ)(1−ω(t))−λ(η−1)            ×(1+e−2iθ)ω(t)]t)−1)dt).

Thus, the assertion (24) of Theorem 3 follows directly from (30).

Next, we derive a convolution property for the class *M*_*m*_^*l*^(*θ*, *λ*, *η*).

**Theorem 4**

Let *ξ* ∈ *C* and |*ξ*| = 1. Then *f* ∈ *M*_*m*_^*l*^(*θ*, *λ*, *η*) if and only if

(31) f∗{(1−ξ)·[1z−∑n=1∞nϕn(α,β;l;m)zn]  +[ξη+ξ(η−1)e−2iθ−1]  ·[1z+∑n=1∞1−λ+nλ1−2λϕn(α,β;l;m)zn]}≠0,                     (z∈U∗).

**Proof**

From the definition (18), we know that *f* ∈ *M*_*m*_^*l*^(*θ*, *λ*, *η*) if and only if

(32) (((eiθ(1−2λ)z(Hml[α,β]f(z))′)  ×((1−λ)Hml[α,β]f(z)+λz(Hml[α,β]f(z))′)−1)  +ηcos⁡θ+isinθ)×((η−1)cos⁡θ)−1≠1+ξ1−ξ,                 (z∈U∗;|ξ|=1),

which is equivalent to

(33) −(1−ξ)z(Hml[α,β]f(z))′+[ξη+ξ(η−1)e−2iθ−1] ·(1−λ)Hml[α,β]f(z)+λz(Hml[α,β]f(z))′1−2λ≠0.

On the other hand, we find from (14) that

(34) −z(Hml[α,β]f(z))′ =1z−∑n=1∞nϕn(α,β;l;m)anzn =f(z)∗[1z−∑n=1∞nϕn(α,β;l;m)zn],(1−λ)Hml[α,β]f(z)+λz(Hml[α,β]f(z))′1−2λ =1z+∑n=1∞1−λ+nλ1−2λϕn(α,β;l;m)anzn =f(z)∗[1z+∑n=1∞1−λ+nλ1−2λϕn(α,β;l;m)zn].

Combining (33) and (34), we get assertion (31) of Theorem 4.

Now, we discuss the coefficient estimates for functions in the class *M*_*m*_^*l*^(*θ*, *λ*, *η*).

**Theorem 5**

Suppose that *f* ∈ *M*_*m*_^*l*^(*θ*, *λ*, *η*). Then

(35) |a1|≤(1−2λ)(η−1)cos⁡θ(1−λ)ϕ1(α,β;l;m),|an|≤(1−2λ)(η−1)cos⁡θ(1−λ)nϕn(α,β;l;m) ×∏k=1n−1(k+1)(1−λ)+2(1−λ+kλ)(η−1)cos⁡θk+2,                    (n∈N∖{1}).

**Proof**

Let *f* ∈ *M*_*m*_^*l*^(*θ*, *λ*, *η*). Then there exists *τ* ∈ *P* such that

(36) eiθ(1−2λ)z(Hml[α,β]f(z))′(1−λ)Hml[α,β]f(z)+λz(Hml[α,β]f(z))′ =(η−1)cos⁡θτ(z)−ηcos⁡θ−isinθ,                (z∈U∗).

It follows from (36) that

(37) eiθ(1−2λ)z(Hml[α,β]f(z))′=[(1−λ)Hml[α,β]f(z)+λz(Hml[α,β]f(z))′] ×[(η−1)cos⁡θτ(z)−ηcos⁡θ−isinθ].

Combining (1) and (37), we have

(38) eiθ(1−2λ)[−1z+∑n=1∞nϕn(α,β;l;m)anzn] =[(1−2λ)1z+∑n=1∞(1−λ+nλ)ϕn(α,β;l;m)anzn]  ·[−eiθ+(η−1)cos⁡θτ1z+(η−1)cos⁡θτ2z2+⋯].

Evaluating the coefficient of *z* ^*n*^ in both sides of (38) yields

(39) 2eiθ(1−λ)ϕ1(α,β;l;m)a1=(1−2λ)(η−1)cos⁡θτ2,eiθ(n+1)(1−λ)ϕn(α,β;l;m)an =(η−1)cos⁡θ[(1−2λ)τn+1    +∑k=1n−1ϕk(α,β;l;m)(1−λ+kλ)akτn−k],(n≥2).

By observing the fact that |*τ*_*n*_| ≤ 2 for *n* ∈ *N*, we find from (39) that

(40) $$\begin{array}{c} \left|a_{1}\right|\leq \frac{\left(1-2\lambda \right)\left(\eta -1\right)\cos\theta }{\left(1-\lambda \right)\phi_{1}\left(\alpha ,\beta ;l;m\right)}, \end{array}$$

(41) |an|≤2(η−1)cos⁡θ(n+1)(1−λ)ϕn(α,β;l;m) ×[1−2λ+∑k=1n−1ϕk(α,β;l;m)(1−λ+kλ)|ak|],(n≥2).

Now we define the sequence {*A*_*n*_}_*n*=1_^*∞*^ as follows:

(42) A1=(1−2λ)(η−1)cos⁡θ(1−λ)ϕ1(α,β;l;m),An+1=2(η−1)cos⁡θ(n+2)(1−λ)ϕn+1(α,β;l;m) ×[1−2λ+∑k=1nϕk(α,β;l;m)(1−λ+kλ)Ak].

In order to prove that

(43) $$\begin{array}{c} \left|a_{n}\right|\leq A_{n}\;\left(n\in N\right), \end{array}$$

we use the principle of mathematical induction. Note that

(44) $$\begin{array}{c} \left|a_{1}\right|\leq A_{1}=\frac{\left(1-2\lambda \right)\left(\eta -1\right)\cos\theta }{\left(1-\lambda \right)\phi_{1}\left(\alpha ,\beta ;l;m\right)}. \end{array}$$

Therefore, assume that

(45) $$\begin{array}{c} \left|a_{k}\right|\leq A_{k}\;\left(k=1,2,\ldots ,n;\;\;n\in N\right). \end{array}$$

Combining (41) and (42), we get

(46) |an+1|≤2(η−1)cos⁡θ(n+2)(1−λ)ϕn+1(α,β;l;m) ×[1−2λ+∑k=1nϕk(α,β;l;m)(1−λ+kλ)|ak|]≤2(η−1)cos⁡θ(n+2)(1−λ)ϕn+1(α,β;l;m) ×[1−2λ+∑k=1nϕk(α,β;l;m)(1−λ+kλ)Ak]=An+1.

Hence, by the principle of mathematical induction, we have

(47) $$\begin{array}{c} \left|a_{n}\right|\leq A_{n}\;\left(n\in N\right), \end{array}$$

as desired. By means of Lemma 2 and (42), we know that (20) holds. Combining (47) and (20), we readily get the coefficient estimates asserted by Theorem 5.

**Remark 6**

By setting *θ* = 0, *l* = 2, *m* = 1, *α*_1_ = *α*_2_ = *β*_1_ = 1, and *λ* = 0 in Theorem 5, we get the corresponding result due to Wang et al. [5].

In what follows, we present some sufficient conditions for functions belonging to the class *M*_*m*_^*l*^(*θ*, *λ*, *η*).

**Theorem 7**

Let *ζ* be a real number with 0 ≤ *ζ* < 1. If *f* ∈ *M* satisfies the condition

(48) |(1−2λ)z(Hml[α,β]f(z))′(1−λ)Hmlf(z)+λz(Hml[α,β]f(z))′+1|≤1−ζ,(z∈U∗),

then *f* ∈ *M*_*m*_^*l*^(*θ*, *λ*, *η*) provided that

(49) $$\begin{array}{c} \cos\theta \geq \frac{1-\zeta }{\eta -1}. \end{array}$$

**Proof**

From (48), it follows that

(50) (1−2λ)z(Hml[α,β]f(z))′(1−λ)Hml[α,β]f(z)+λz(Hml[α,β]f(z))′  =−1+(1−ζ)ω(z),

where *ω* is analytic in *U* with *ω*(0) = 0 and |*ω*(*z*)| < 1. Thus, we have

(51) R(eiθ(1−2λ)z(Hml[α,β]f(z))′(1−λ)Hml[α,β]f(z)−λz(Hml[α,β]f(z))′) =R(eiθ(−1+(1−ζ)ω(z))) =−cos⁡θ+(1−ζ)R(eiθω(z)) ≥−cos⁡θ−(1−ζ)|ω(z)| >−cos⁡θ−(1−ζ) ≥−ηcos⁡θ,

provided that cos⁡*θ* ≥ (1 − *ζ*)/(*η* − 1). This completes the proof of Theorem 7.

If we take *ζ* = 1 − (*η* − 1)cos⁡*θ* in Theorem 7, we obtain the following result.

**Corollary 8**

If *f* ∈ *M* satisfies the inequality

(52) |(1−2λ)z(Hml[α,β]f(z))′(1−λ)Hml[α,β]f(z)+λz(Hml[α,β]f(z))′+1| ≤(η−1)cos⁡θ,

then *f* ∈ *M*_*m*_^*l*^(*θ*, *λ*, *η*).

**Theorem 9**

If a function *f* ∈ *M* given by (1) satisfies the inequality

(53) ∑n=1∞[(1−λ)(1+n)
sec
θ+(η−1)(1−λ+nλ)]  ×ϕn(α,β;l,m)|an|≤(1−2λ)(η−1),

then it belongs to the class *M*_*m*_^*l*^(*θ*, *λ*, *η*).

**Proof**

In virtue of Corollary 8, it suffices to show that condition (52) holds. We observe that

(54) |(1−2λ)z(Hml[α,β]f(z))′(1−λ)Hml[α,β]f(z)+λz(Hml[α,β]f(z))′+1| =(1−λ)  ×|∑n=1∞(1+n)ϕn(α,β;l,m)anzn+11−2λ+∑n=1∞(1−λ+nλ)ϕn(α,β;l,m)anzn+1| <(1−λ)∑n=1∞(1+n)ϕn(α,β;l,m)|an|1−2λ−∑n=1∞(1−λ+nλ)ϕn(α,β;l,m)|an|.

The last expression is bounded by (*η* − 1)cos⁡*θ*, if

(55) (1−λ)∑n=1∞(1+n)ϕn(α,β;l,m)|an| ≤(η−1)cos⁡θ[1−2λ         −∑n=1∞(1−λ+nλ)ϕn(α,β;l,m)|an|],

which is equivalent to

(56) ∑n=1∞[(1−λ)(1+n)sec θ+(η−1)(1−λ+nλ)]  ×ϕn(α,β;l,m)|an|≤(1−2λ)(η−1).

This completes the proof of Theorem 9.
